# [¹¹C]Methionine PET uptake kinetics in corticotroph pituitary neuroendocrine tumors

**DOI:** 10.1038/s41598-026-39219-7

**Published:** 2026-02-27

**Authors:** Anthime Flaus, Armand Pattée, Guillaume Criton, Nicolas Costes, Elise Levigoureux, Julie Haesebaert, Alexandre Vasiljevic, IMPEC Collaborators, Claire Briet, Frederic Castinetti, Justine Cristante, Delphine Drui, Natacha Germain, Luigi Maione, Frederic Illouz, Emmanuel Sonnet, Igor Tauveron, Ines Merida, Sophie Lancelot, Lucien Marchand, Sylvie Rode, Mireille Bertholon-Gregoire, Véronique Lapras, Emmanuel Jouanneau, François Cotton, Claire Bournaud, Gerald Raverot

**Affiliations:** 1 Service de Médecine Nucléaire , Hospices Civils de Lyon, Lyon, France; 2 Lyon Neuroscience Research Center, CNRS UMR5292, INSERM U1028, Université Claude Bernard Lyon 1, Lyon, France; 3 Radiology Department, Hospices Civils de Lyon, Centre Hospitalier Lyon Sud, Lyon, France; 4 Laboratoire CREATIS, CNRS UMR 5220, Inserm U1296, INSA Lyon, Villeurbanne, France; 5 CERMEP-Life Imaging, Lyon, France; 6 Radiopharmacy Department, Hospices Civils de Lyon, Groupement Hospitalier Est, Bron, France; 7 Research on Healthcare Performance (RESHAPE), Université Claude Bernard Lyon 1, INSERM, Lyon, France; 8 Service de Recherche et d’épidémiologie Cliniques, Pôle de Santé Publique, Hospices Civils de Lyon, Lyon, France; 9 Department of Neuropathology, Hospices Civils de Lyon, Bron, France; 10 Skull Base and Pituitary Unit, Department of Neurosurgery B, Neurological Hospital, Lyon, France; 11 Inserm U1052, CNRS UMR5286, Cancer Research Center of Lyon, Lyon, France; 12 Université Claude Bernard Lyon 1, Villeurbanne, France; 13 Centre Hospitalier de Roanne, Service d’Endocrinologie, Roanne, France; 14 CNRS UMR5286, INSERM, U1052, Cancer Research Center of Lyon, Lyon, France; 15 Endocrinology Department, Reference Center for Rare Pituitary Diseases HYPO, “Groupement Hospitalier Est” Hospices Civils de Lyon, Bron, France; 16 Department of Endocrinology, Reference Center for Rare Pituitary Diseases HYPO, Angers University Hospital, Angers, France; 17 MITOVASC, CarMe Team, CNRS UMR 6015, INSERM U1083, Angers University, Angers, France; 18 Department of Endocrinology, Centre de Référence des Maladies Rares hypophysaires HYPO, INSERM, UMR1251, Marseille Medical Genetics, Institut MarMaRa, Hôpital La Conception, APHM, Aix Marseille Univ, Marseille, France; 19 CHU Grenoble Alpes, INSERM UMR 1292, CEA, IRIG Biosanté, Univ Grenoble Alpes, Grenoble, France; 20 Department of Endocrinology Diabetology and Nutrition, L’institut du thorax, Nantes University, CHU de Nantes, Nantes, France; 21 Department of Endocrinology, Diabetes, Metabolism and Eating Disorders, University Hospital of Saint-Etienne, Saint-Etienne, France; 22 TAPE Research Group, Eating Disorders, Addictions & Extreme Bodyweight, Jean Monnet University, Saint-Etienne, France; 23 Université Paris-Saclay, Inserm UMR-S1185, Physiologie et Physiopathologie Endocriniennes, APHP, Hôpital Bicêtre, Service d’Endocrinologie et des Maladies de la Reproduction, Le Kremlin-Bicêtre, France; 24 CHRU Brest, Service diabétologie-endocrinologie, Brest, France; 25 Service d’Endocrinologie, Diabétologie et Maladies Métaboliques, CHU Clermont-Ferrand, Clermont-Ferrand, France; 26 Laboratoire GReD, Université Clermont Auvergne, Clermont-Ferrand, France; 27 Service d’Endocrinologie, Hôpital Saint Joseph Saint Luc, Lyon, France; 28 Centre de consultation de la Sauvegarde, Lyon, France; 29 Clinique du Renaison, Roanne, France; 30 Hospices Civils de Lyon, Centre Hospitalier Lyon Sud, Service de Radiologie, Lyon, France

**Keywords:** Amino-acid, Molecular imaging, Adenoma, PET/MRI, Cushing’s disease, Dynamic analysis, Biomarkers, Cancer, Endocrinology

## Abstract

[^11^C]Methionine ([^11^C]MET) PET provides high sensitivity to localize corticotroph pituitary neuroendocrine tumors (PitNETs) in *de novo* Cushing’s disease but remains inconclusive in up to 20% of cases. Current PET protocols rely on late acquisitions (20–40 min post-injection). Since other endocrine tumors present earlier peak tracer uptake, corticotroph PitNETs might also exhibit early differential amino acid uptake, compared to the normal pituitary gland. This exploratory study based on data derived from a previously registered prospective multicenter cohort study (ClinicalTrials.gov identifier: NCT03346954) included 15 patients with pathologically confirmed corticotroph PitNETs accurately localized using MRI. Kinetic analysis showed rapid early uptake in PitNETs and normal pituitary gland, followed by a significant decline over time, with consistently higher uptake in PitNETs (*p* < 0.05), but no statistically significant difference in temporal uptake pattern (*p* = 0.09). Early uptake slope and peak uptake were significantly higher in PitNETs compared to normal gland (*p* < 0.01), with moderate-to-high discriminative performance (area under the curve 0.78; 95%CI 0.60–0.94 and 0.86; 95%CI 0.69–0.98, respectively). Time-to-peak showed no statistically significant discriminative value. These findings suggest that kinetic parameters of [¹¹C]MET PET, particularly early slope and peak uptake, may provide complementary information to static PET for PitNET characterization.

**Supplementary Information:**

The online version contains supplementary material available at 10.1038/s41598-026-39219-7.

## Introduction

Pituitary neuroendocrine tumor (PitNET)/pituitary adenoma of corticotroph type causes Cushing’s disease (CD), a rare endocrine disorder^1,2^. Accurate tumor localization is essential for curative surgery^3^. Magnetic resonance imaging (MRI) is the imaging diagnostic method of choice although its sensitivity is approximately 70% for corticotroph PitNETs^4^. This limitation frequently leads to inconclusive workup or to perform exploratory surgery, which result in a risk of incomplete resection and persistent disease^3^. Molecular imaging, using radiolabeled amino acid (AA) positron emission tomography (PET), such as [^11^C]Methionine ([^11^C]MET) PET, provides high sensitivity; a prospective study reported a sensitivity similar to that of 3T MRI to assess the accurate localization of small corticotroph PitNETs in de *novo* CD^6^.

The current [^11^C]MET PET imaging protocols are derived from those used in gliomas with a fixed start time at 20 min after injection^6^. In other endocrine tumors, such as parathyroid adenomas, an early uptake pattern (e.g. between 2 and 9 min after injection) has been described^7,8^. Therefore, our hypothesis is that corticotroph PitNETs could also present early differential AA uptake compared to the normal pituitary gland, which could be detected through earlier PET imaging. In addition, on dynamic contrast-enhanced MRI, an early differential enhancement was observed, in which marked PitNET image contrast appears within the first seconds^9,10^.

The primary aim of the study was to characterize the early uptake kinetics of [^11^C]MET PET in PitNETs and in the normal pituitary gland, and to explore whether simple kinetics parameters may provide complementary discriminatory value to differentiate the PitNET from the normal gland in *de novo* CD. The secondary aim was to explore the sensitivity of early static [^11^C]MET PET images to tumor localization compared with standard late imaging (20–40 min).

## Materials and methods

### Study design and patients

This study is an exploratory ancillary analysis based on data derived from a previously registered prospective multicenter cohort study (ClinicalTrials.gov identifier: NCT03346954)^5^. The studies were performed in line with the principles of the Declaration of Helsinki. The former study was approved by the Ile de France VIII Ethics Committee (EudraCT: 2017–002721–38). The ancillary study was approved by the institutional medical ethics committee of Hospices Civils de Lyon (August 5, 2025; Ref: 25-5284). Informed consent was obtained from all participants in accordance with French MR-004 regulations; under this framework, patients are informed and may oppose the reuse of their data, with written consent required only in cases of opposition. This ancillary analysis was restricted to MRI–positive cases to ensure accurate volume-of-interest (VOI) definition and was designed primarily to characterize [¹¹C]MET uptake kinetics rather than to optimize tumor localization, particularly in MRI negatives cases.

Consecutive patients were included when the following criteria were met: ≥18 years old with *de novo* CD, corticotroph PitNETs confirmed through pathological analysis and accurately localized using MRI, and with dynamic PET data available.

### Data collection

The following clinical and imaging data were retrieved from medical records and previous analyses^5^: age, sex, medical treatment, tumor secretory activity (serum adrenocorticotropic hormone [ACTH] and 24-hour urinary free cortisol [UFC]), MRI and surgical localization (left/right/median), MRI-derived tumor volume, late PET localization, and pathological confirmation of corticotroph PitNETs.

### Image acquisition and reconstruction

Imaging was performed on a 3.0-Tesla PET/MRI hybrid scanner (Biograph mMR Siemens)^11^. PET list-mode acquisition began immediately before intravenous bolus injection of 363 ± 90 MBq [^11^C]MET and lasted 40 min.

Images were reconstructed using a 3D ordinary Poisson ordered-subset expectation maximization algorithm with 3 iterations of 21 subsets. Data correction (normalization, attenuation, and scatter correction) was fully integrated within the reconstruction process. Gaussian post-reconstruction filtering (full width at half maximum = 4 mm) was applied. Reconstructions were performed with a zoom of 2 yielding a voxel size of 2.08 × 2.08 × 2.03 mm^3^ in a matrix of 172 × 172 × 127 voxels.

For kinetic analysis, images were reconstructed into 6 frames of 20 s and 27 frames of 60 s. Early static images were reconstructed using acquisition from 20 s to 2 min post-injection. This time window was selected empirically based on inspection of time–activity curve, corresponding to the phase of maximal uptake increase before washout, and to align with time window reported for dynamic MRI^9,10,12,13^. Late static images were reconstructed using acquisition from 20 to 40 min after injection as reference. Details regarding [^11^C]MET synthesis, static PET reconstruction, and MRI sequences are available in Flaus et al.^6^.

### Images analysis

For the kinetic PET image analysis, mean standardized uptake values (SUVs) were measured for each time frame using Inveon Research Workplace 4.2 (Siemens). VOIs were defined on MRI by a neuroradiologist: one covering the PitNET and one covering the normal pituitary gland (Figure S1). Three kinetic parameters were derived from time–activity curves for each VOI. Time-to-peak was defined as the time point of maximum SUVmean. Peak SUVmean was defined as the maximum SUVmean value at time-to-peak. Early uptake slope was calculated as the difference between the peak SUVmean and the SUVmean at the first frame, divided by the corresponding time interval.

Static early PET images analyses were performed using Syngo.via Software (Siemens) by 2 readers blinded to the clinical data in a randomized order. A PitNET was considered present when the MET uptake was greater than that of the surrounding normal pituitary gland^14^; otherwise, PET was classified as negative. The MET uptake localization was categorized as left, right, or median. In case of discordance, a consensus was reached through concomitant review of the two readers.

### Study endpoints

The primary endpoint was the quantitative characterization of early [¹¹C]MET uptake kinetics in corticotroph PitNETs and the normal pituitary gland. Specifically, we aimed: (1) to compare the temporal changes in SUVmean between PitNETs and the normal pituitary gland using a linear mixed-effects model; and (2) to evaluate whether kinetic-derived parameters (time-to-peak, early uptake slope, and peak SUVmean) provide discriminative value to differentiate PitNETs from normal gland, using pathological confirmation as reference.

Secondary endpoints included: (1) comparison of the sensitivity of early static [¹¹C]MET PET images with conventional late images (20–40 min) for PitNET localization; and (2) comparison of clinical and imaging characteristics between correctly and incorrectly localized PitNETs on early PET.

### Statistical analysis

Statistical analyses were performed using Python 3.12 (https://www.python.org). Continuous variables are expressed as median and interquartile range (IQR), and categorical variables as counts and percentages.

To evaluate differences in uptake kinetics between PitNETs and the normal pituitary gland, a linear mixed-effects model was applied with SUVmean as the dependent variable, time (continuous), tissue type (gland vs. PitNET used as the reference), and their interaction as fixed effects, and the patient as a random effect.

Kinetic-derived parameters—including time-to-peak, early uptake slope, and peak SUVmean—were compared between tissues using the Mann–Whitney U test.

The discriminative performance of kinetic parameters to differentiate PitNET from normal pituitary gland was assessed using receiver operating characteristic (ROC) analysis. Area under the curve (AUC) values with 95% confidence intervals were estimated using non-parametric bootstrap resampling.

The sensitivity of PET for correct tumor localization was calculated using pathological analysis as the reference standard, with two-sided 95% confidence intervals estimated using the exact binomial method. Localization performance between early and late PET images was compared using McNemar’s test. Patient characteristics were compared between correctly and incorrectly localized PitNETs using Fisher’s exact test for categorical variables and the Mann–Whitney U test for continuous variables.

A two-sided p-value < 0.05 was considered statistically significant. Given the rarity of corticotroph PitNETs and the exploratory nature of dynamic PET analyses, this study was designed to generate hypotheses rather than to provide definitive diagnostic thresholds and all analyses were considered exploratory.

## Results

### Patients

**Fig. 1:**
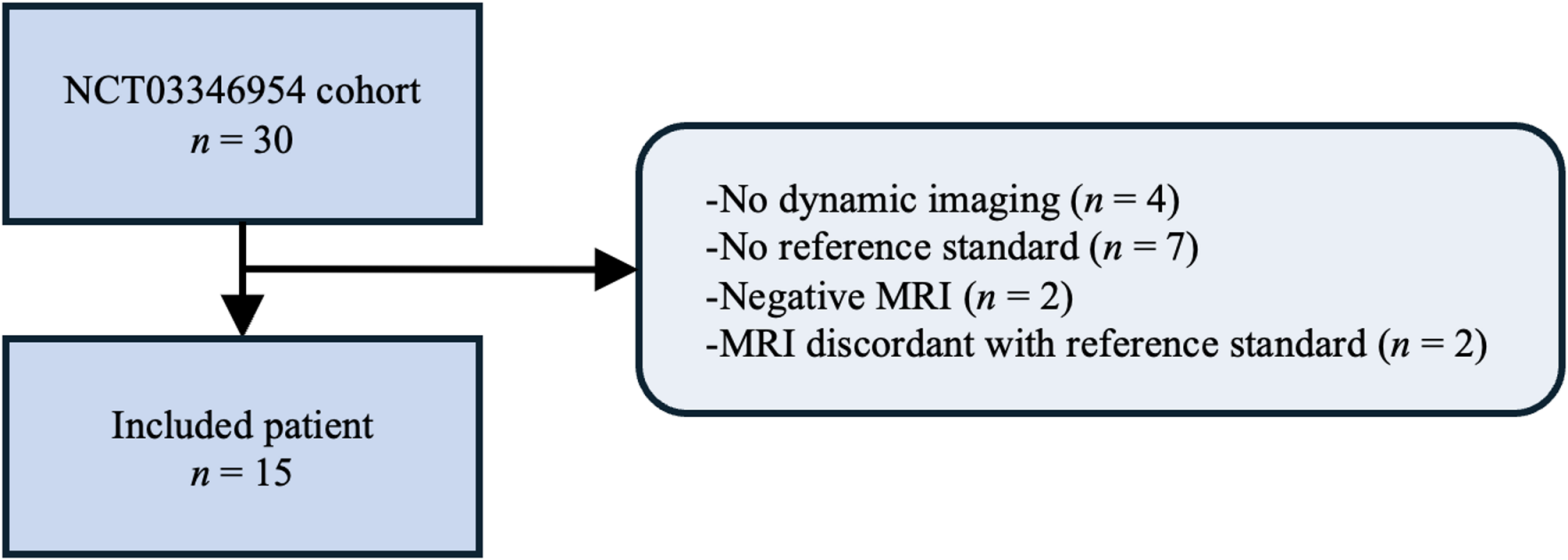
Flow diagram of patient inclusion.

Fifteen patients were included (Fig. 1). Overall, 12 patients were female (80%), median age was 46 years (IQR 36–51), and median weight was 88.1 kg (IQR 73.5-109.9). At inclusion, median 24 h UFC was 2.6 (IQR 1.4–5.1) times above the upper limit of normal (ULN) and median ACTH was 1 (IQR 0.6–1.3) times above the ULN. Among all patients, 6 (40%) received adrenal steroidogenesis inhibitors before surgery (ketoconazole in 3 [20%], osilodrostat in 1 [7%], mitotane in 1 [7%], and metyrapone in 1 [7%]). MRI localized the tumor at the right side in 6 (40%), the median in 3 (20%), and the left side in 6 (40%) cases, and results were confirmed by the pathological analysis. The median MRI-derived tumor volume was 62.5mm^3^ (IQR 45.5–147). The median Ki67 was 2.3% (IQR 1-3.7).

### Uptake kinetics of [¹¹C]MET in PitNETs and in the normal pituitary glands

**Fig. 2:**
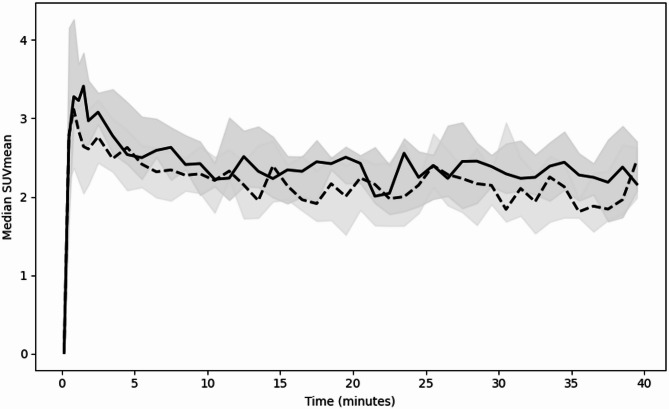
Time-activity curves of median SUVmean in PitNETs (solid regression line) and normal pituitary gland (dashed line). SUV: Standardized uptake value, Shaded areas represent the interquartile range.

Kinetic analysis showed a rapid early uptake of MET in both PitNETs and the normal pituitary gland (Fig. 2). The linear mixed-effects model confirmed a statistically significant decline of SUVmean over time (β = -0.012, *p* < 0.0001) and consistently higher SUVmean values in PitNETs than in the normal gland (β = -0.409, *p* = 0.01). However, the non-statistically significant interaction term (β = 0.005, *p* = 0.09) indicating that although PitNETs exhibited higher uptake, their temporal uptake pattern did not differ meaningfully from that of normal gland.

The early uptake slope was significantly different between PitNETs and normal pituitary gland (median 5.17 [IQR 3.3–7.7] vs. 2.6 [IQR 0.9–4.5], *p* < 0.01) and provided moderate to good discriminative result (AUC 0.78, 95%CI [0.60–0.94]). Peak SUVmean was significantly higher in PitNETs (median 4.30 [IQR 3.9–5.3] vs. 3.45 [IQR 3.2–3.7]; *p* < 0.0001) with a strong discriminative performance (AUC 0.86, 95%CI [0.69–0.98]. In contrast, time-to-peak did not differ between tissues (median 0.8 [IQR 0.5-1.0] vs. 1.2 [IQR 0.7-3.0]; *p* = 0.15) and showed no discriminative value (AUC 0.35, 95%CI [0.15–0.55]).

### Sensitivity of early PET acquisitions

**Fig. 3:**
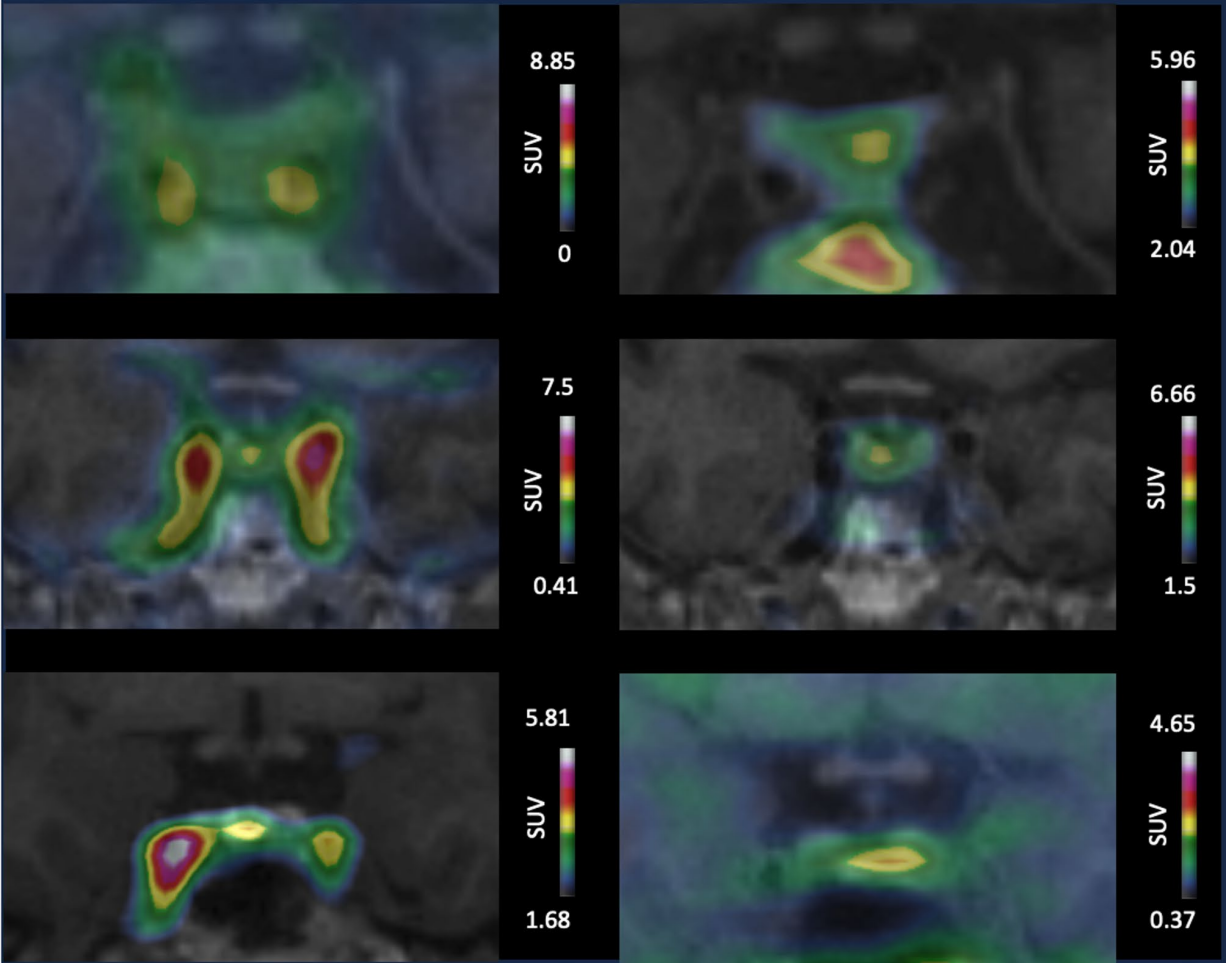
Early and late superimposed [^11^C] MET PET/MRI images for PitNET localization. First column shows early coronal [^11^C]MET PET/MRI images. Second column displays corresponding late [^11^C]MET PET/MRI images. First row: Patient 11 presents no focal uptake on early image but left-sided focal uptake on late image confirmed by pathology. Second row: Patient 10 presents clear right focal uptake, concordant on early and late images confirmed by pathology. Third row: Patient 7 presents clear right-sided focal uptake on early image confirmed by pathology, which was discordant from a left focal uptake on late images. MET: Methionine, SUV: standardized uptake values, PitNET: pituitary neuroendocrine tumors.

Results of the visual analysis regarding PitNETs localization are presented for early and late time frames in Table S1. No statistically significant difference was found between early (10/15; 66%; 95%CI [0.38;0.88]) compared to late images (12/15; (80%; 95%CI [0.52;0.96]) (*p* = 0.38). One case was identified only on early images and three cases identified only on late images. Examples of concordant and discordant cases are illustrated in Fig. 3.

The subgroup comparison between correctly and incorrectly localized PitNETs using early [^11^C]MET PET images found not statistically significant difference in terms of sex, age, MRI-derived tumor volume, Ki67 index, UFC and ACTH serum level, use of treatment, Ki67 index, and correct dynamic MRI localization (Table S2).

## Discussion

This study provided the first characterization of [¹¹C]MET uptake kinetics in corticotroph PitNETs in patients with *de novo* CD. Corticotroph PitNETs demonstrated a significantly steeper early uptake slope and higher peak SUVmean than the normal gland. These parameters showed moderate-to-high discriminative performance, suggesting that kinetic-derived parameters may provide complementary information beyond static imaging.

From a clinical interpretation standpoint, these kinetic differences may be used to increase reader confidence by supporting the biological PitNET related origin of focal uptake, as PitNET detection often relies on subtle differences in uptake intensity against a background of physiologically active pituitary tissue^14,15^. In this context, kinetic-derived parameters such as early uptake slope or peak SUVmean may help confirm the PitNET origin of a focal uptake already suspected on static images, particularly in small lesions or in borderline tumor-to-gland contrast. This observation is clinically relevant, as prior studies demonstrated that [¹¹C]MET PET results can influence surgical decision-making in patients with CD and equivocal or negative MRI findings^16,17^.

Additionally, these findings offer insight into the biological behavior of corticotroph PitNETs. Of note dynamic contrast-enhanced MRI showed that the normal pituitary gland typically enhances earlier, within the first seconds, and more intensely due to its dense vascularization and absence of a blood–brain barrier, followed by signal washout whereas PitNETs show delayed enhancement with maximal contrast occurring within the first two to three minutes^9,10^. The discordance between vascular MRI dynamics and [¹¹C]MET PET kinetics supports the concept that amino-acid PET reflects tumor-specific biological processes, potentially related to amino-acid transport and metabolic activity rather than perfusion alone.

Despite these kinetic differences, early [¹¹C]MET PET acquisitions did not improve PitNET localization compared to conventional late imaging. Several factors may explain this finding. First, the rapid early uptake observed in both PitNETs and normal pituitary tissue likely limits lesion-to-background contrast during the initial minutes after injection. Second, short early frames are affected by lower count statistics, reducing signal-to-noise ratio and impairing visual detectability. Third, the small size and heterogeneous uptake of corticotroph PitNETs make them particularly susceptible to partial-volume effects, especially during early acquisitions. In contrast, late images benefit from more stable tumor-to-background ratios and higher count statistics, resulting in more robust and reliable visual localization.

Several limitations should be acknowledged. First, the small sample size limits statistical power, although it reflects the rarity of CD and leverages the only prospective cohort published to date with combined pathological confirmation and dynamic [¹¹C]MET PET data. Second, the analysis focused on simplified kinetic parameters (time-to-peak, early uptake slope, and peak SUVmean) chosen for their feasibility in routine clinical practice. More advanced kinetic modeling, potentially requiring arterial input functions and larger dataset, could provide deeper mechanistic insight but was beyond the scope of this study. Third, the inclusion of only MRI-positive cases may limit generalizability to MRI-negative CD, where molecular imaging is often most clinically impactful however, this restriction was necessary to allow accurate VOI definition.

Despite these limitations, the present findings support the relevance of dynamic [¹¹C]MET PET acquisition for corticotroph PitNET characterization. Kinetic parameters may complement conventional static imaging by providing quantitative confirmation of PitNET-related uptake. Importantly, these results lay the groundwork for future studies using formal kinetic modeling approaches, such as reference-tissue graphical analysis or voxel-wise parametric mapping, and for integration with advanced analytical methods including machine learning. Larger prospective studies are required to validate these approaches and to define the clinical role of quantitative kinetic analysis of amino-acid PET in CD.

## Conclusion

Analysis of [¹¹C]MET PET revealed distinct uptake kinetics between corticotroph PitNET and normal pituitary tissue. In particular, early uptake slope and peak uptake showed moderate-to-high discriminative performance, suggesting they may provide complementary information to static PET for PitNET characterization.

## Supplementary Information

Below is the link to the electronic supplementary material.

Supplementary Material 1

## Data Availability

The datasets generated and/or analysed during the current study are not publicly available due to health data regulation but are available from the corresponding author on reasonable request.

## Abbreviations

AA: Amino acid
ACTH: Adrenocorticotropic hormone
CD: Cushing’s disease
IQR: Interquartile range
MET: Methionine
MRI: Magnetic resonance imaging
PET: Positron emission tomography
PitNET: Pituitary neuroendocrine tumors
SUV: Standardized uptake values
UFC: Urinary free cortisol
ULN: Upper limit of normal
VOI: Volumes of interest
