# Supplementary material for: [¹¹C]Methionine PET uptake kinetics in corticotroph pituitary neuroendocrine tumors
Source: Sci Rep. 2026 Feb 27;16:8854. doi: 10.1038/s41598-026-39219-7 (PMC12988236; doi:10.1038/s41598-026-39219-7)
Supplement: Supplementary file 1 — Supplementary Material 1 [file 41598_2026_39219_MOESM1_ESM.docx]

**SUPPLEMENTARY DATA**

**TABLE S1** C-11 MET PET and pathological findings for corticotroph PitNETs localization

| Patient | Early PET | Late PET | Pathological analysis |
| --- | --- | --- | --- |
| 1 | left | left | left |
| 2 | median | left | left |
| 3 | median | left | left |
| 4 | left | left | left |
| 5 | median | median | median |
| 6 | left | left | right |
| 7 | right | left | right |
| 8 | right | right | median |
| 9 | right | right | right |
| 10 | right | right | right |
| 11 | left | left | left |
| 12 | median | median | median |
| 13 | no target | right | right |
| 14 | left | left | left |
| 15 | right | right | right |

PitNET: pituitary neuroendocrine tumors, MET: Methionine

**TABLE S2** Characteristics of patients according to the correct or incorrect localization of the PitNET by early [^11^C] MET PET

|  | Correctly localized with early PET  N=10 | Incorrectly localized with early PET  N=5 |  | p-values |  |
| --- | --- | --- | --- | --- | --- |
| Sex (Female), n (%) | 8 (80%) | 4 (80%) |  | 1.0 |  |
| Age (years) | 41 (34-48) | 50 (49-54) |  | 0.11 |  |
| UFC (xULN) | 3.4 (2.3-5.4) | 1.4 (1.3-1.8) |  | 0.22 |  |
| ACTH (xULN) | 0.9 (0.6-1.4) | 1.1 (0.9-1.1) |  | 0.68 |  |
| Treatment, n (%) | 5 (50%) | 1 (20%) |  | 0.31 |  |
| MRI-derived tumor volume (mm^3^) | 55.3 (44.8-117) | 70 (47.3-208) |  | 0.58 |  |
| Correct dynamic MRI localization, n (%)* | 8 (80%) | 3 (60%) |  | 0.56 |  |
| Ki67 index (%) | 2.7 (1.3-3.9) | 2.3 (1-2.6) |  | 0.67 |  |

Data are expressed as median (interquartile range), unless otherwise specified. UFC: urinary free cortisol, ACTH: adrenocorticotropic hormone, xULN: times above upper limit of normal, PitNET: pituitary neuroendocrine tumors, MET: methionine. *using the localization of the surgical specimen as a reference, after pathologic confirmation of PitNET of the corticotroph type.


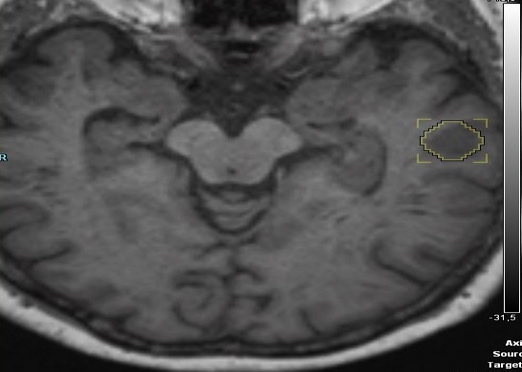

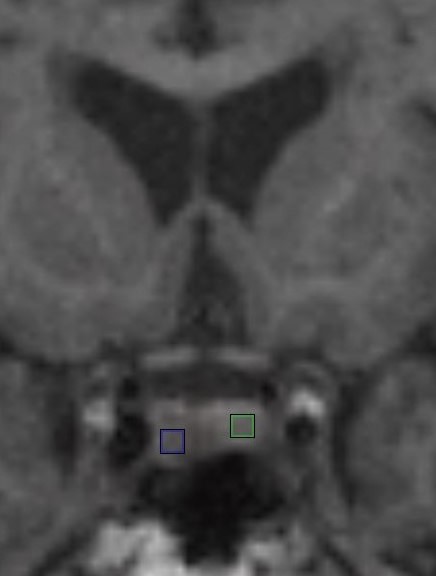


-31.5

735

FIGURE S1: Example of MRI with volume of interest in the adenoma (dark blue), the normal pituitary gland (green).

Analyses were performed using Inveon Research Workplace 4.2 Siemens. Volumes of interest (VOI) were defined on the co-registered MRI transferred to dynamic PET frames. Their size was 20 voxels. Adenoma VOI was manually corrected when necessary.
